# Serum neurofilament light chain: a predictive marker for outcomes following mild-to-moderate ischemic stroke

**DOI:** 10.3389/fneur.2024.1398826

**Published:** 2024-05-22

**Authors:** Chongxi Xu, Tong Yi, Ting Qing, Yongliang Jiang, Xingyang Yi, Jianguo Xu, Junpeng Ma

**Affiliations:** ^1^Department of Neurosurgery, West China Hospital, Sichuan University, Chengdu, Sichuan, China; ^2^Department of Neurology, West China Hospital, Sichuan University, Chengdu, Sichuan, China; ^3^Department of Neurology, The Second People’s Hospital of Deyang City, Deyang, Sichuan, China; ^4^Department of Neurology, People’s Hospital of Deyang City, Deyang, Sichuan, China

**Keywords:** serum neurofilament light chain protein, ischemic stroke, early neurological deterioration, functional outcomes, cardiovascular events

## Abstract

**Background:**

Biomarkers that reflect brain damage or predict functional outcomes may aid in guiding personalized stroke treatments. Serum neurofilament light chain (sNfL) emerges as a promising candidate for fulfilling this role.

**Methods:**

This prospective, observational cohort investigation included 319 acute ischemic stroke (IS) patients. The endpoints were the incidence of early neurological deterioration (END, an elevation of two or more points in the National Institute of Health stroke scale score within a week of hospitalization compared with the baseline) and functional outcome at 3 months (an mRS score of >2 at 3 months was categorized as an unfavorable/poor functional outcome). The association of sNfL, which was assessed within 24 h of admission, with END and unfavorable functional outcomes at follow-up was assessed via multivariate logistic regression, whereas the predictive value of sNfL for unfavorable functional outcomes and END was elucidated by the receiver operating characteristic curve (ROC).

**Results:**

Of 319 IS individuals, 89 (27.90%) suffered from END. sNfL not only reflects the severity of stroke measured by NIHSS score (*p* < 0.05) but also closely related to the severity of age-related white matter changes. Higher initial NIHSS score, severe white matter lesions, diabetes mellitus, and upregulated sNfL were significant predictors of END. Similarly, the multivariate logistic regression analysis results showed that elevated sNfL, a higher baseline NIHSS score, and severe white matter lesions were substantially linked with unfavorable outcomes for 3 months. Similarly, sNfL was valuable for the prediction of the 3 months of poor outcome (95%CI, 0.504–0.642, *p* = 0.044). Kaplan–Meier analysis shows that patients with elevated sNfL levels are more likely to reach combined cerebrovascular endpoints (log-rank test *p* < 0.05).

**Conclusion:**

This investigation suggests that sNfL can serve as a valuable biomarker for predicting END and 3-month poor functional outcomes after an IS and has the potential to forecast long-term cardiovascular outcomes.

## Introduction

Stroke is a global disease characterized by a high incidence and disability and mortality rates (1, 2). Given the substantial global burden imposed by ischemic stroke (IS), it becomes imperative to identify a circulating biomarker that can serve as a reflection of brain injury (3). Neurofilament light chain (NfL) is a highly expressed neuronal cytoplasmic protein in large-caliber myelinated axons (4), and its concentration in both cerebrospinal fluid (CSF) and blood significantly increases during neuronal damage (5, 6). Although recent studies have shown that levels of NfL in blood are influenced by other confounding factors such as patient age, blood volume, body mass index (BMI), and renal function (7), and elevated levels are also observed in non-primary neurological disorders, for example, elevated levels of NfL have been observed in COVID-19 patients during the acute phase, correlating with clinical severity and adverse outcomes (8). However, its application as a biomarker of neuronal axonal injury in patients with primary neurological diseases profoundly changes current diagnostic and prognostic approaches to neurological disorders. Consequently, assessing NfL levels in cerebrospinal fluid or serum holds great potential for diagnosing, prognosticating, and monitoring neurologic disorders (4, 5). The literature has indicated that increased NfL levels are linked with the severity of Alzheimer’s disease (9), multiple sclerosis (10), amyotrophic lateral sclerosis (11), etc. Much research has focused on the role of NfL in cerebrovascular disease, particularly IS. Tiedt et al. (12). demonstrated that ischemic stroke (IS) individuals have higher serum NfL (sNfL) levels than healthy individuals. Uphaus et al. found a clear link between sNfL levels and the degree of functional outcome after IS. Overall, evidence suggests that measuring sNfL can offer valuable prognostic insights into long-term cardiovascular outcomes in patients with IS.

Early neurological deterioration (END) is a frequently observed manifestation of the acute phase after IS (9, 10), and the incidence of which varies ranging from 12 to 42% (13). Additionally, the prognosis of IS patients with END is poor (12, 14). However, few studies have investigated the association of sNfL with END after IS. NfL level increases with age (15), and advanced age is substantially linked with substandard prognosis in IS patients (16). Therefore, whether the predictive value of sNfL in IS patients was affected by age remains unclear. Moreover, small vessel disease has been confirmed to be related to the recurrence of strokes in acute IS patients (17, 18). Whether sNfL can be a biomarker for small vessel disease-induced white matter lesions (WMLs) still requires validation. This investigation defined END as an elevation of two or more points in NIHSS score within a week after admission (17). Moreover, this investigation aimed to elucidate the association of upregulated sNfL with END occurrence and long-term prognosis in patients with IS.

## Materials and methods

### Patients

**Figure 1:**
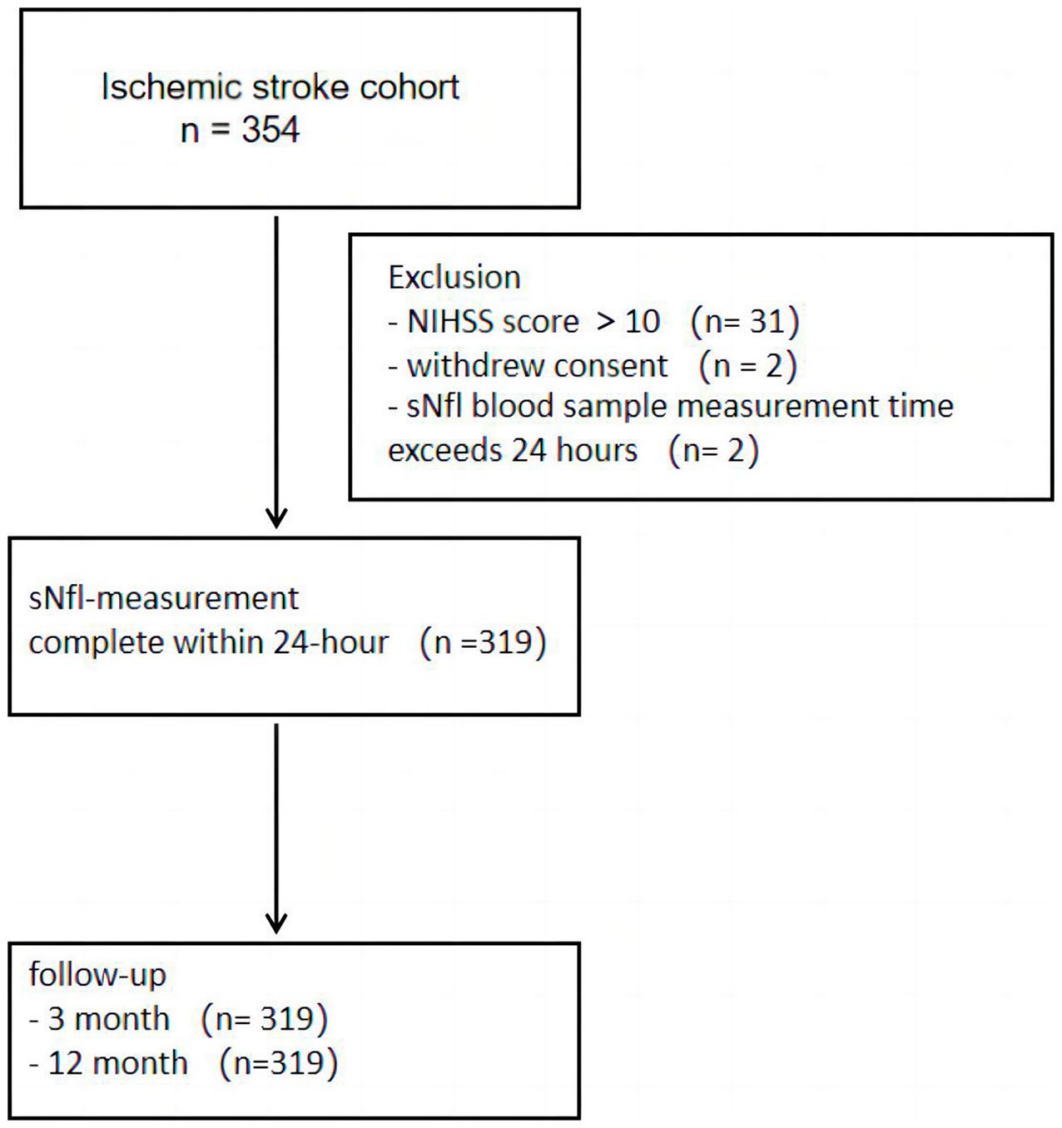
A flowchart depicting participant’s enrollment/follow-up.

Between March 2019 and July 2021, 319 IS patients, confirmed through symptoms and imaging findings, were admitted to multiple centers within 48 h after the onset of symptoms. The data of partial patients were obtained from a prospective observational study (19). These individuals were selected for this investigation if they were ≥18 years of age and had National Institutes of Health Stroke Scale (NIHSS) score between 1 and 10. Individuals with pre-stroke modified Rankin Scale (mRS) score > 2, transient ischemic attack (TIA), and who were undergoing endovascular thrombectomy, had severe liver or kidney dysfunction or cardiac impairment, coexistence of other severe systemic diseases, concurrent diagnosis of malignant neoplasms, active antitumor therapy, other neurodegenerative disorders, or autoimmune conditions affecting the nervous system, were pregnant, lactating, or had the potential for pregnancy (including those planning to conceive), and those expressing unwillingness to participate in the research were excluded. Figure 1 indicates the patient’s Consolidated Standards of Reporting Trials (CONSORT) diagram.

This investigation was authorized by the ethical board of the participating hospitals. All the participants were first informed about the research, and then, their consent was acquired. This research was registered in the Chinese Clinical Trial Registry with registration number ChiCTR2000029902.

### Data collection and clinical evaluation

Demographic data (gender, age), patient’s medical history (including hypertension, diabetes, hyperlipidemia, atrial fibrillation, and coronary heart disease), Glasgow Coma Scale (GCS) and NIHSS scores at admission, baseline blood pressure, and random blood glucose, followed by fasting venous blood tests for lipid profile, glycated hemoglobin, coagulation parameters, and routine biochemical markers. All patients underwent thorough CT/MRI examinations, and further CTA or DSA examinations were performed based on specific clinical indications and registration of patient’s hospital treatment, including thrombolysis, blood pressure and sugar management, statin use, and antithrombotic therapy. The sNfL levels were assessed within 24 h of admission via enzyme-linked immunoassay (ELISA; Supplementary Methods) (20). The WMLs were assessed semi-quantitatively using a modified scale that specifically designed for age-related white matter changes (ARWMC rating) (21).

### Clinical outcome variables

The primary endpoint was END, defined as a gradual decline in neurological function, represented by an elevation of two or more points in the NIHSS total score within a week of hospitalization compared with the baseline. An elevation of ≥1 point in the motor items of the NIHSS was observed, excluding cases with hemorrhagic transformation after cerebral infarction, recurrent new cerebral infarction, cerebral edema, and worsening caused by severe organ dysfunction.

The secondary endpoint was the functional prognosis of patients 90 days after onset, assessed by the mRS. An mRS score of ≤2 at 3 months was considered a favorable functional outcome, while that of >2 was categorized as an unfavorable/poor functional outcome.

Other outcomes included new composite vascular events of recurrent IS, TIA, intracranial hemorrhage (ICH), and mortality during 12 months of follow-up.

### Statistics

Continuous variables are depicted as mean ± standard deviation. The normality of numerical variable was elucidated via the Kolmogorov–Smirnov test. For comparing normally distributed variables, a two-tailed independent group t-test was applied. Variables which not normally distributed were analyzed by the Mann–Whitney test. For categorical variables, percentages were used, and a chi-square test was carried out for comparison. In cases of minimal predicted variable frequencies, Fisher’s exact test was carried out. Univariate analysis methods were used to investigate patients’ clinical data and baseline characteristics with and without END. Risk factors for END and poor 3-month outcome (mRS > 2) were explored through multivariate logistic stepwise regression analysis. The receiver operating characteristic (ROC) curve was applied to assess the ability of sNfL to predict END and a poor 3-month outcome. Survival function estimates for clinical outcomes based on the level of sNfL were elucidated via the Kaplan–Meier curve and log-rank test. Survival curves were acquired at 12 months. *p*-value < 0.05 was termed statistically important.

## Results

**Figure 2:**
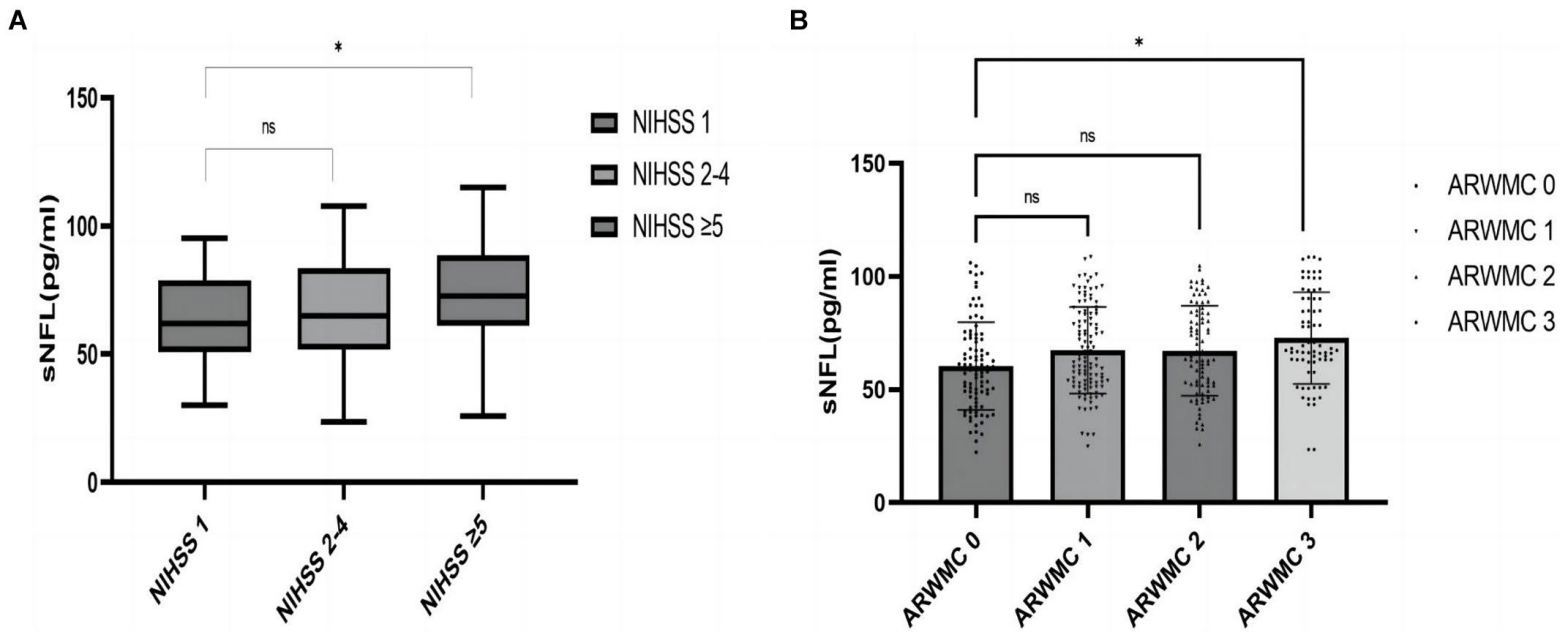
**(A)** Stratified analysis elucidated the association of sNfL (serum neurofilament light chain) levels with stroke severity (NIHSS score). **(B)** Stratified analysis evaluated the relationship between sNfL levels and age-related white matter changes. ^*^p-value < 0.05.

This investigation enrolled 319 acute IS patients; 89 (27.90%) of these suffered from END. Diabetes mellitus was more common, and clinical deficits were more severe on admission (initial NIHSS score, *p* = 0.005), and the level of sNfL was higher (*p* = 0.029) in patients with END than those without END according to univariate analysis (Supplementary Table 1). Furthermore, MRI/CT imaging revealed that WMLs were more severe (ARWMC Rating 2 and 3) in END patients (*p* < 0.05; Supplementary Table 1). The significant increase in sNfL expression is correlated with the severity of stroke [NIHSS 1: sNFL median, 63.98 (IQR, 50.78–78.81) pg./ml; NIHSS 2–4: sNFL median, 67.45 (IQR, 51.70–83.65) pg./mL; NIHSS ≥ 5: sNFL median, 73.87 (IQR, 61.06–88.58) pg./mL; ANOVA *p* = 0.0067, Figure 2A]. No correlation was observed between the age of IS patients and the expression of sNfL [age < 50 years: sNFL median, 70.71 (IQR, 58.84–78.74); age 50–59 years: sNFL median, 67.18 (IQR, 50.78–84.90); age 60–69 years: sNFL median, 62.95 (IQR, 50.55–78.60); age ≥ 70 years: sNFL median, 66.57 (IQR, 51.12–83.89); ANOVA *p* = 0.3143] (Supplementary Figure 1). However, a marked rise was indicated in sNfL levels with severe WMLs than its absence [ARWMC scale 3: sNFL median, 72.82 (IQR, 62.39–90.29) pg./mL; ARWMC 0: sNFL median, 60.41 (IQR, 46.84–72.73) pg./mL; *p* = 0.0004] (Figure 2B). Moreover, there is no significant correlation between NfL levels and patients’ history of prior atrial fibrillation, as well as the onset of new atrial fibrillation following hospital admission (*p* > 0.05).

Following multivariate analysis, several variables were determined to be closely linked with the incidence of END after IS, including initial NIHSS score [odds ratio (OR), 1.186, with a 95% confidence interval (CI), 1.069–1.317 (*p* = 0.001)], sNfL (OR, 1.014; 95% CI, 1.001–1.028; *p* = 0.040), diabetes mellitus (OR, 2.160; 95% CI, 1.104–4.227, *p* = 0.025), ARWMC scale 2 (OR, 3.587; 95% CI, 1.617–7.958, *p* = 0.002), and ARWMC scale 3(OR, 3.075; 95% CI, 1.153–8.202, *p* = 0.025; Table 1). These findings indicate that a higher NIHSS score, severe white matter hyperintensities, diabetes mellitus, and upregulated sNfL are significant predictors of END after IS. After their discharge, all the patients were followed up. The functional outcome of each patient was assessed at 3 months using the mRS score, and those with an mRS score of >2 were considered to have a poor functional prognosis. Upon conducting univariate analysis, several factors were identified as potentially associated with poor outcome, including older age, elevated sNfL levels, lower GCS and higher NIHSS scores at admission, and the presence of white matter hyperintensities. These findings are shown in Supplementary Table 2. The multivariate logistic regression results indicated that severe white matter hyperintensities, elevated sNfL, and a higher NIHSS score at admission were strongly correlated with poor functional outcomes (Table 2).

**Table 1:** Multivariate logistic regression analysis with regard to the early neurological deterioration after stroke.

Variable	OR	95%CI	*p*-value
Diabetes mellitus	2.160	1.104–4.227	0.025^*^
Serum NfL value	1.014	1.001–1.028	0.040^*^
Blood glucose at admission	1.043	0.980–1.110	0.187
NIHSS score at admission	1.186	1.069–1.317	0.001^*^
ARWMC(2)	3.587	1.617–7.958	0.002^*^
ARWMC(3)	3.075	1.153–8.202	0.025^*^

ARWMC (Age-Related White Matter Changes) Rating Scale 2, Beginning confluence of lesions; ARWMC Rating Scale 3, diffuse involvement of the entire region, with or without involvement of U fibers. ^*^P-value < 0.05.

**Table 2:** Multiple logistic regression with 3-month poor functional outcome (mRS > 2).

Variable	OR	95%CI	*P*-value
Age	1.026	0.999–1.053	0.059
Serum NfL value	1.015	1.001–1.029	0.038^*^
GCS score at admission	0.938	0.780–1.129	0.501
NIHSS score at admission	1.271	1.136–1.422	<0.001^*^
ARWMC(2)	3.038	1.333–6.924	0.008^*^
ARWMC(3)	4.424	1.661–11.781	0.003^*^

ARWMC (Age-Related White Matter Changes) Rating Scale 2, Beginning confluence of lesions; ARWMC Rating Scale 3, diffuse involvement of the entire region, with or without involvement of U fibers. ^*^P-value < 0.05.

**Figure 3:**
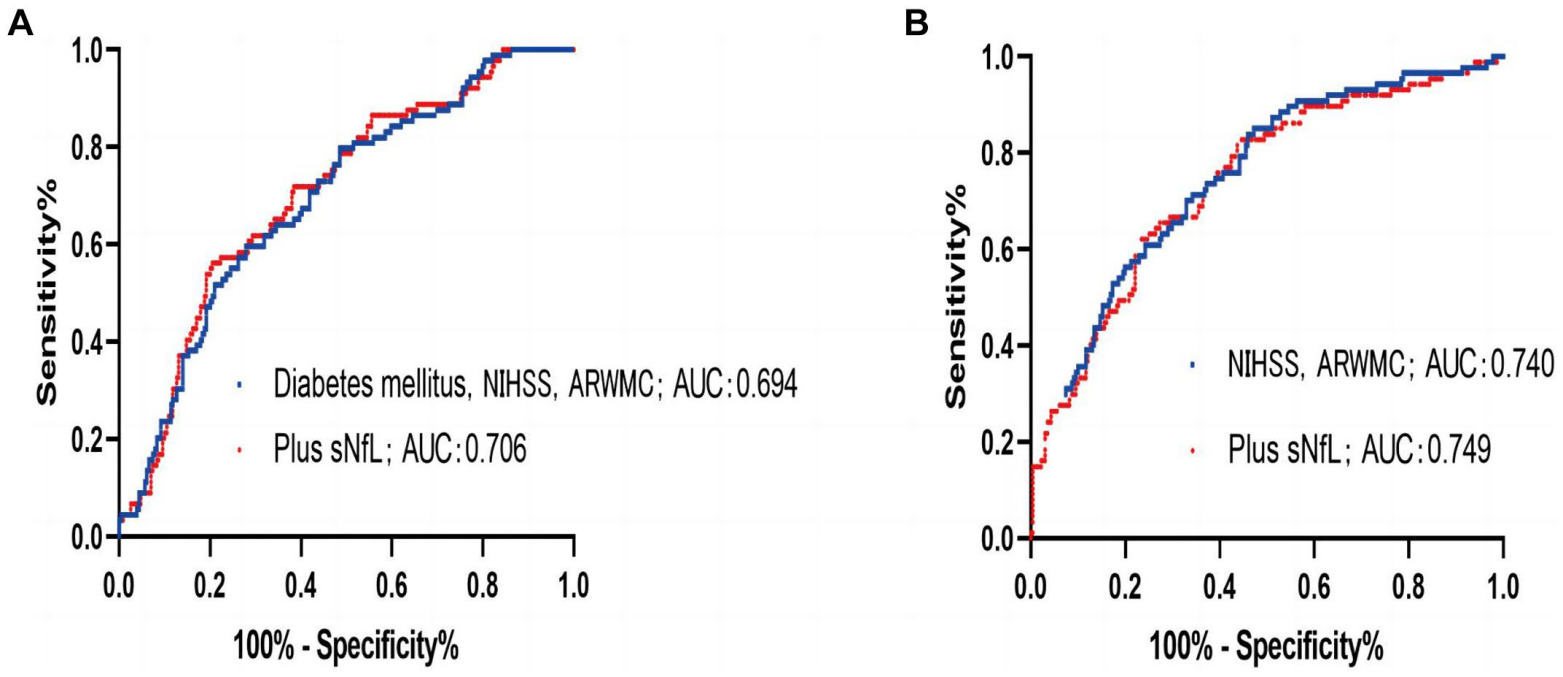
**(A)** ROC to predict the early neurological deterioration for the combination of diabetes mellitus, National Institutes of Health Stroke Scale (NIHSS), and ARMWC-rating (blue) exhibited an area under the curve (AUC) of 0.694 (95% CI, 0.631–0.756; *p* < 0.001). The additional use of sNfL (red) showed a trend toward an independent improvement of AUC to 0.706 (95% CI, 0.644–0.767; *p* < 0.001). **(B)** ROC to predict the 3 months of poor outcome for the combination of National Institutes of Health Stroke Scale (NIHSS) and ARMWC-rating (blue) exhibited an area under the curve (AUC) of 0.740 (95% CI, 0.678–0.801; *p* < 0.001). The additional use of sNfL (red) showed a trend toward an independent improvement of AUC to 0.749 (95% CI, 0.690–0.809; *p* < 0.001).

The ROC was employed using the sNfL for predicting END development odds within IS cases. The optimal threshold point was 53.40, its sensitivity was 83.1%, and its specificity was 36.5%. The AUC was 0.586 (95% CI, 0.519–0.653, *p* = 0.017). Moreover, the combination of variables including sNfL, initial NIHSS score, and ARWMC rating increased the AUC ≤0.706 (95% CI, 0.644–0.767; *p* < 0.001; Figure 3A). Through ROC analysis, the predictive value of sNfL on poor outcome at 3 months was analyzed, the optimal threshold point was 62.09, its sensitivity was 63.6%, and its specificity was 51.1%. The AUC was 0.573 (95% CI, 0.504–0.642, *p* = 0.044). Similarly, the receiver operating curve was drawn for variables that remain valid after multivariate analysis, and the predictive value of combination of variables including sNfL, NIHSS score, and ARWMC rating on poor outcome at 3 months is shown in Figure 3B, and the AUC was 0.749 (95% CI, 0.690–0.809, *p* < 0.001).

**Figure 4:**
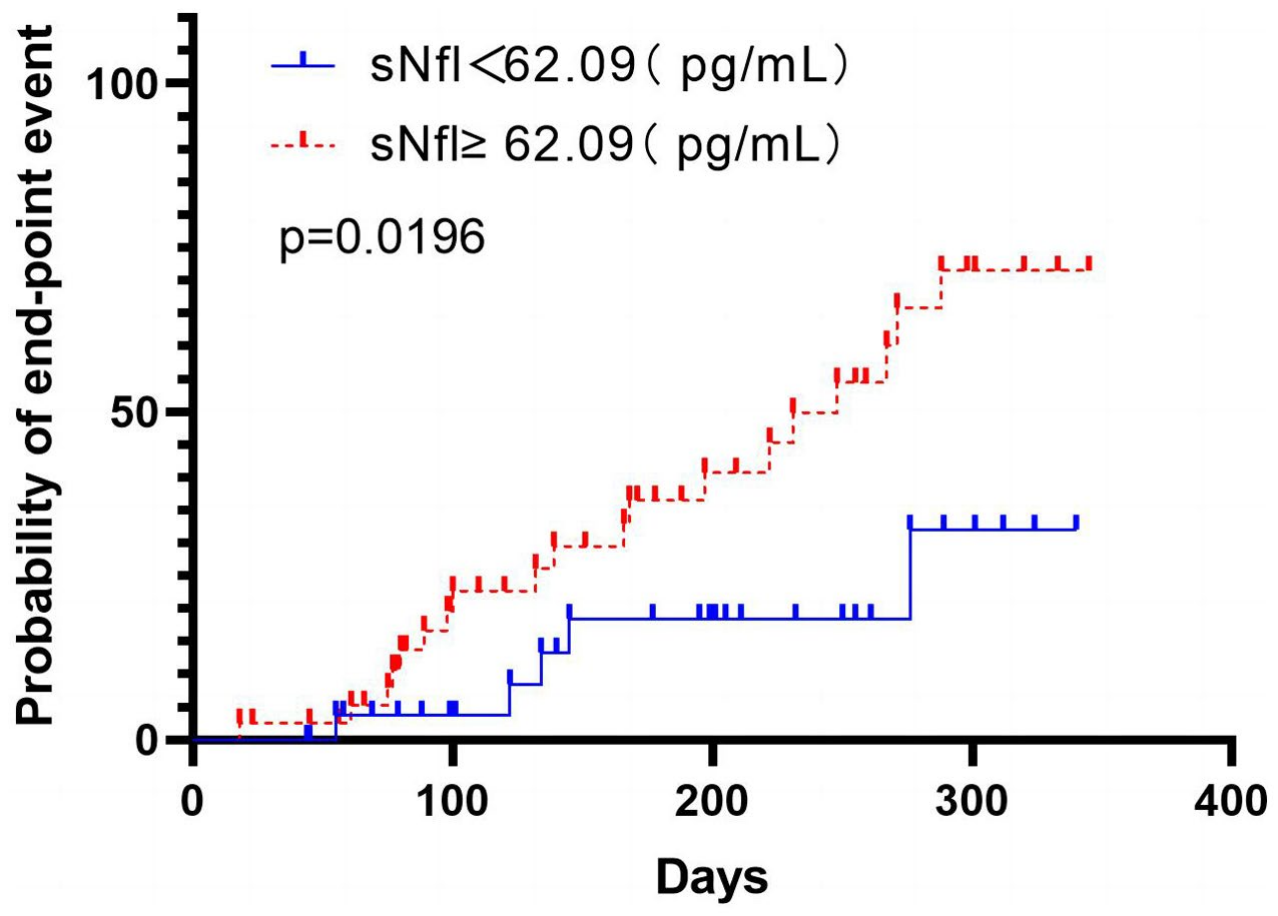
The time to cardiovascular endpoint (recurrent IS, intracranial hemorrhage, and mortality) was analyzed according to the division of patients using the Youden index based on the sNfL level’s ROC curve analysis. Patients with high sNfL levels (≥62.09 pg./mL) are represented in red, while those with low sNfL levels (<62.09 pg./mL) are represented in blue. The analysis was performed using non-logarithmic-transformed values, and the intergroup differences were assessed via the log-rank test. The data indicated markedly significant intergroup differences (*p* = 0.0196), indicating that patients with high sNfL levels had a shorter time to reach the cardiovascular endpoint than those with low NfL levels.

Test characteristics of sNfL ≥ 62.09 pg./mL based on the Youden Index for prediction of poor functional outcome (mRS > 2) at 3-month after the index event. According to the Kaplan–Meier analysis, there was a markedly higher incidence of composite vascular events in participants with high sNfL levels during the 12-month follow-up (*p* = 0.0196), including recurrent IS, ICH, and mortality (Figure 4).

## Discussion

The reported (2016) lifetime risk of stroke in adults is approximately 25% globally (22). END is a frequent comorbidity associated with acute IS, and its occurrence can have significant implications for both short-term outcomes, such as morbidity and death, and long-term recovery from stroke (17). Although the definition of END remains variable (11, 23), many studies have reported its relatively high incidence from 12% to 42% (19, 24). This study defined END as an increase of two or more points in NIHSS score within a week after admission. Here, it was observed that the incidence of END was 27.9%. Moreover, it was indicated that sNfL, HNISS score, diabetes, and severe white matter lesions (ARWMC Rating Scales 2 and 3) were independently related to the occurrence of END. Additionally, using the ROC curve analysis, upregulated sNfL indicated significant predictive value for IS patients who experienced END and showed a trend of independent improvement in AUC in joint diagnosis, highlighting its potential as a reliable predictive factor.

The NfL is a primary constituent and backbone of neurofilaments (25), essentially involved in axonal growth, stability, and intracellular transport of substances (4). It has high specificity for neuronal injury and death. Under normal conditions, NfL remains relatively stable, with low levels in peripheral blood. However, during neuronal damage, its expression increased, making it a valuable biomarker for neuronal injury. Initially, we hypothesized that sNfL is a biomarker for neuronal axonal injury, with its serum levels associated with age and clinical deficits following IS. However, the obtained data indicate a correlation between sNfL levels and the clinical deficits during hospitalization (assessed by NIHSS) but is independent of the age of IS patients. This discovery is inconsistent with previous research results. There might be several reasons for this inconsistency, for instance, the findings by Traenka et al. (26) were limited to cervical artery dissection patients, and blood samples were measured within 30 days. The study by Marcis included TIA patients and used a modified electrochemiluminescence (ECL) immunoassay, and blood samples were drawn upon admission or after 24 h (27). Moreover, Uphaus et al. (28) and Sellner et al. (29) also indicated different results on the correlation between neurofilament levels and the NIHSS score. However, Sellner et al. used neurofilament heavy chains for research. It is noteworthy that sNfL levels can be influenced by the blood collection timings (30). From the data of this investigation, Nfl may reflect central nervous system injury, as its levels correlate with the severity of stroke (NIHSS score). Therefore, even in the cases of transient ischemic attack and negative neuroimaging, Nfl levels may still provide evidence of central nervous system injury. This underscores its significance as an important adjunct for assessing and treating various types of stroke patients. While Nfl levels are correlated with age (7), our results indicate that in ischemic stroke patients, advanced age and atrial fibrillation do not show a significant correlation with Nfl levels.

This investigation categorized ARWMC into four levels based on the research by Wahlund (21). A substantial difference was indicated in sNfL levels between patients classified as severe white matter lesions (ARWMC Rating Scale 3) and those classified as no lesions (ARWMC Rating Scale 0). The data were consistent with previous findings, as a correlation was depicted between sNfL and white matter hyperintensities (25, 26), suggesting that sNfL might act as an index for chronic and ongoing neuroaxonal damage. Furthermore, the binary logistic regression analysis discovered that severe white matter lesions (ARWMC Rating Scales 2 and 3) were independently associated with END.

Remarkably, it was found that upregulated sNfL, initial NIHSS score, and ARWMC Rating Scales 2 and 3 were also independently related to the 3-month poor mRS score, which was consistent with the literature on IS (22, 27). The ROC curve data indicated that while the sensitivity may be lower compared with the initial HNISS score prediction, the sNfL value can still be a reliable indicator for predicting a poor outcome for 3 months. Considering that sNfL depicts ongoing chronic axonal damage and the chronic progression of small vessel disease (30–34), the participants were followed up for 12 months. The follow-up endpoint events included recurrent IS, ICH, and mortality. Kaplan–Meier analysis indicated a markedly higher hazard for endpoint within a median 12-month follow-up in patients with >62.09 pg./mL sNfL values, which was measured within 24 h after the stroke, further supporting the notion that upregulated sNfL indicate a greater likelihood for IS patients to experience worsening of their early and long-term outcome symptoms.

There are several limitations to this study. First, END evaluation requires the exclusion of other factors that can increase NIHSS scores, such as hemorrhagic transformation, brain edema, or systemic diseases. Since patients with NIHSS scores > 10 upon admission were excluded, there may be a selection bias in the study population. Second, the 12-month follow-up period and the inclusion of various endpoint events, such as recurrent IS, ICH, and mortality, have also limited this investigation. Although we conducted a second sNfL measurement for patients experiencing cardiovascular events, there were instances where some patients did not undergo the second serum sNfL test, and the results were not available. Therefore, the association of immediate sNfL levels with recurrent cardiovascular events remains unclear.

## Conclusion

Overall, the acute IS patients indicated a high occurrence of END. Furthermore, higher initial NIHSS score, severe white matter hyperintensities, upregulated sNfL, and the presence of diabetes were independently linked with END. Additionally, a higher initial NIHSS score, severe white matter hyperintensities, and upregulated sNfL were also independent variables linked with poor functional outcomes for 3 months. The data indicated that sNfL could be a reliable indicator for predicting END and poor functional outcomes and might also help identify individuals with an enhanced risk for cardiovascular events, making it a potential predictor for future therapeutic trials.

## Data availability statement

The original contributions presented in the study are included in the article/Supplementary material, further inquiries can be directed to the corresponding author.

## Ethics statement

This investigation was authorized by the Ethical Board of the Deyang Peoples' Hospital. All the participants were first informed about the research, and then, their consent was acquired. The studies were conducted in accordance with the local legislation and institutional requirements. The participants provided their written informed consent to participate in this study.

## Author contributions

CX: Conceptualization, Data curation, Investigation, Methodology, Resources, Supervision, Validation, Writing – original draft, Writing – review & editing. TY: Conceptualization, Investigation, Methodology, Resources, Software, Validation, Writing – original draft, Writing – review & editing. TQ: Conceptualization, Data curation, Formal analysis, Methodology, Resources, Validation, Writing – original draft. YJ: Formal analysis, Methodology, Project administration, Validation, Writing – review & editing. XY: Data curation, Formal analysis, Project administration, Resources, Validation, Writing – review & editing. JX: Funding acquisition, Investigation, Supervision, Validation, Writing – original draft, Writing – review & editing. JM: Conceptualization, Formal analysis, Investigation, Methodology, Project administration, Software, Supervision, Validation, Visualization, Writing – original draft, Writing – review & editing.

## Supplementary material

SUPPLEMENTARY FIGURE 1 Stratified analysis was conducted to evaluate the relationship between sNfL (neurofilament light chain) levels and age.
