# Supplementary figures and images for: Serum neurofilament light chain: a predictive marker for outcomes following mild-to-moderate ischemic stroke
Source: Front Neurol. 2024 May 22;15:1398826. doi: 10.3389/fneur.2024.1398826 (PMC11150679; doi:10.3389/fneur.2024.1398826)

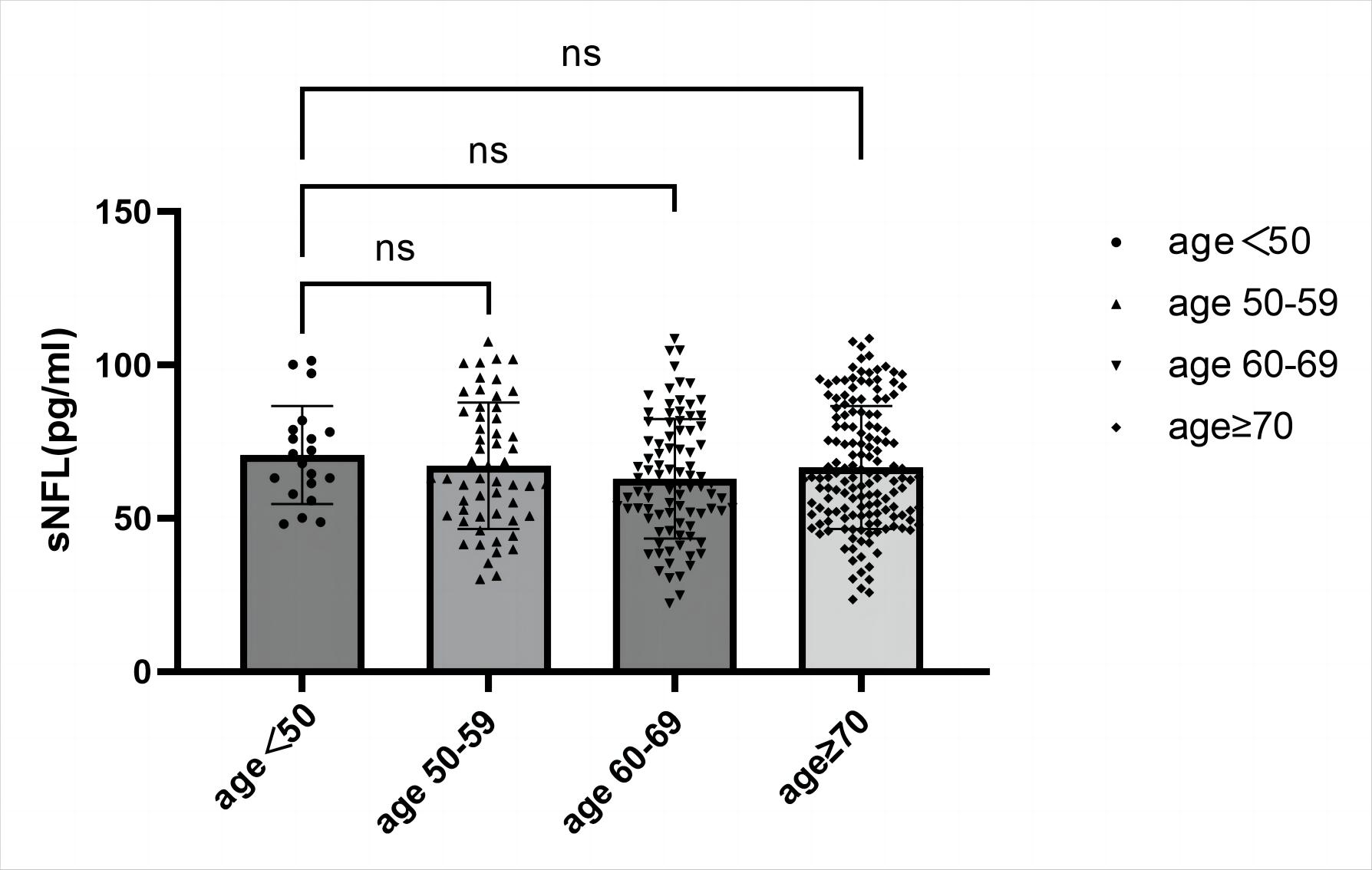

Supplement: SUPPLEMENTARY FIGURE 1 — Stratified analysis was conducted to evaluate the relationship between sNfL (neurofilament light chain) levels and age. [file Image_1.JPEG]
