# Supplementary material for: Serum neurofilament light chain: a predictive marker for outcomes following mild-to-moderate ischemic stroke
Source: Front Neurol. 2024 May 22;15:1398826. doi: 10.3389/fneur.2024.1398826 (PMC11150679; doi:10.3389/fneur.2024.1398826)
Supplement: Supplementary file 2 [file Data_Sheet_1.docx]

Serum Neurofilament Light Chain: A Predictive Marker for Outcomes following Mild to Moderate Ischemic Stroke

Chongxi Xu^1†^, Tong Yi^2†^, Ting Qing^3^, Yongliang Jiang^1^, Xingyang Yi^4^, Jianguo Xu^1^, Junpeng Ma^1^*****

^1^ Department of Neurosurgery, West China Hospital, Sichuan University, No. 37, Guoxue Alley, Wuhou District, Chengdu, Sichuan, 610000, China. Email: [xuchongxi@stu.scu.edu.cn](mailto:xuchongxi@stu.scu.edu.cn).

^2^ Department of Neurology, West China Hospital, Sichuan University, No. 37, Guoxue Alley, Wuhou District, Chengdu, Sichuan, 610000, China. Email: yitong948664yt@163.com

^3^ Department of Neurology, The Second People’s Hospital of Deyang City, No. 340 Minjiang West Road, DeYang, Sichuan,618000, China. Email: [qt18783874891@163.com](mailto:qt18783874891@163.com).

^4^ Department of Neurology, People’s Hospital of Deyang City, No.173 TaiShan North Road, DeYang, Sichuan,618000, China. Email: [yixingyang64@126.com](mailto:yixingyang64@126.com).

***** Correspondence author: Junpeng Ma

Department of Neurosurgery, West China Hospital of Sichuan University, No. 37, Guoxue Alley, Wuhou District, Chengdu, Sichuan, 610000, China. Email: [Majunpeng@scu.edu.cn](mailto:Majunpeng@scu.edu.cn). Fax: 028-85422489.

† These authors contributed equally to this work and share first authorship

**Abstract:**

**Background:** Biomarkers that reflect brain damage or predict functional outcomes may aid in guiding personalized stroke treatments. Serum neurofilament light chain (sNfL) emerges as a promising candidate for fulfilling this role.

**Methods:** This prospective, observational cohort investigation included 319 acute IS patients. The endpoints were the incidence of early neurological deterioration (END, an elevation of 2 or more points in the National Institute of Health stroke scale score within a week of hospitalization compared with the baseline) and functional outcome at 3 months (An mRS score of ＞ 2 at 3 months was categorized as an unfavorable/poor functional outcome). The association of sNfL, which were assessed within 24 hours of admission, with END and unfavorable functional outcomes at follow-up was assessed via multivariate logistic regression, whereas the predictive value of sNfL for unfavorable functional outcomes and END was elucidated by the receiver operating characteristic curves (ROC).

**Results:** Of 319 IS individuals, 89 (27.90%) suffered from END. sNfL not only reflects the severity of stroke measured by NIHSS score (P<0.05), but also closely related to the severity of age-related white matter changes. Higher initial NIHSS score, severe white matter lesions, diabetes mellitus, and upregulated sNfL were significant predictors of END. Likewise, the multivariate logistic regression analysis results showed that elevated sNfL, a higher baseline NIHSS score, and severe white matter lesions were substantially linked with unfavorable outcomes at 3 months. Similarly, sNfL was valuable for the prediction of the 3 months poor outcome (95%CI, 0.504-0.642, p=0.044). Kaplan Meier analysis shows that patients with elevated sNfL levels are more likely to reach combined cerebrovascular endpoints (log-rank test *P*<0.05).

**Conclusions:** This investigation suggests that sNfL can serve as a valuable biomarker for predicting END and 3-month poor functional outcomes after an IS and has the potential to forecast long-term cardiovascular outcomes.

**Key Words:** Serum neurofilament light chain protein; Ischemic stroke; Early neurological deterioration; Functional outcomes; Cardiovascular events.

**Introduction**

Stroke is a global disease characterized by a high incidence and disability and mortality rates[1][2]. Given the substantial global burden imposed by ischemic stroke (IS), it becomes imperative to identify a circulating biomarker that can serve as a reflection of brain injury[3]. Neurofilament light chain (NfL) is a highly expressed neuronal cytoplasmic protein in large-caliber myelinated axons[4], and its concentration in both cerebrospinal fluid (CSF) and blood significantly increases during neuronal damage[5][6]. Although recent studies have shown that levels of NfL in blood are influenced by other confounding factors such as patient age, blood volume, body mass index (BMI), renal function, etc.,[7] and elevated levels are also observed in non-primary neurological disorders, for example, elevated levels of NfL have been observed in COVID-19 patients during the acute phase, correlating with clinical severity and adverse outcomes[8]. However, its application as a biomarker of neuronal axonal injury in patients with primary neurological diseases profoundly changes current diagnostic and prognostic approaches to neurological disorders. Consequently, assessing NfL levels in cerebrospinal fluid or serum holds great potential for diagnosing, prognosticating, and monitoring neurologic disorders[4][9]. The literature has indicated that increased NfL levels are linked with the severity of Alzheimer's disease[10], multiple sclerosis[11], amyotrophic lateral sclerosis[12], etc. Much research has focused on the role of NfL in cerebrovascular disease, particularly IS. Tiedt *et al*[13]. demonstrated that IS individuals have higher serum NfL (sNfL) levels than healthy individuals. Uphaus *et al.* found a clear link between sNfL levels and the degree of functional outcome after IS. Overall, evidence suggests that measuring sNfL can offer valuable prognostic insights into long-term cardiovascular outcomes in patients with IS.

Early neurological deterioration (END) is a frequently observed manifestation of the acute phase after IS[14][15] , the incidence of which varies, ranging from 12% to 42%[16]. Additionally, the prognosis of IS patients with END is poor[17][18]. However, few studies have investigated the association of sNfL with END after IS. NfL level increases with age[19], and advanced age is substantially linked with substandard prognosis in IS patients[20]. Therefore, whether the predictive value of sNfL in IS patients was affected by age remains unclear. Moreover, small vessel disease has been confirmed to be related to the recurrence of strokes in acute IS patients[21][22]. Whether sNfL can be a biomarker for small vessel disease-induced white matter lesions (WMLs) still requires validation. This investigation defined END as an elevation of 2 or more points in NIHSS score within a week after admission[17]. And this investigation aimed to elucidate the association of upregulated sNfL with END occurrence and long-term prognosis in patients with IS.

**Materials and Methods**

**Patients**

Between March 2019 and July 2021, 319 IS patients, confirmed through symptoms and imaging findings, were admitted to multiple centers within 48 hours after the onset of symptoms. The data of partial patients were obtained from a prospective observational study[23]. These individuals were selected for this investigation if they were ≥ 18 years of age and had National Institutes of Health Stroke Scale (NIHSS) score between 1 to 10. Individuals: with pre-stroke modified Rankin Scale (mRS) score＞2, transient ischemic attack (TIA), who were undergoing endovascular thrombectomy, had severe liver or kidney dysfunction or cardiac impairment, coexistence of other severe systemic diseases, concurrent diagnosis of malignant neoplasms, active antitumor therapy, other neurodegenerative disorders, or autoimmune conditions affecting the nervous system, were pregnant, lactating, or had the potential for pregnancy (including those planning to conceive), and those expressing unwillingness to participate in the research were excluded. Figure 1 indicates the patient’s CONSORT (Consolidated Standards of Reporting Trials) diagram.

This investigation was authorized by the ethical board of the participating hospitals. All the participants were first informed about the research, and then their consent was acquired. This research was registered in the Chinese Clinical Trial Registry with registration number ChiCTR2000029902.

**Data Collection and Clinical Evaluation**

Demographic data (gender, age), patient's medical history (including hypertension, diabetes, hyperlipidemia, atrial fibrillation, coronary heart disease), Glasgow Coma Scale (GCS) and NIHSS scores at admission, baseline blood pressure and random blood glucose, followed by fasting venous blood tests for lipid profile, glycated hemoglobin, coagulation parameters, and routine biochemical markers. All patients underwent thorough CT/MRI examinations, and further CTA or DSA examinations were performed based on specific clinical indications, and registration of patient’s hospital treatment, including thrombolysis, blood pressure and sugar management, statin use, and antithrombotic therapy. The sNfL levels were assessed within 24 hours of admission *via* enzyme-linked immunoassay (ELISA) (Supplemental methods)[24]. The WMLs were assessed semi-quantitatively using a modified scale specifically designed for age-related white matter changes (ARWMC rating)[25].

**Clinical Outcome Variables**

The primary endpoint was END, defined as a gradual decline of neurological function, represented by an elevation of 2 or more points in the NIHSS total score within a week of hospitalization compared with the baseline. Or an elevation of ≥1 point in the motor items of the NIHSS was observed. And excluding cases with hemorrhagic transformation after cerebral infarction, recurrent new cerebral infarction, cerebral edema, and worsening caused by severe organ dysfunction.

The secondary endpoint was the functional prognosis of patients 90 days after onset, assessed by the mRS. An mRS score of ≤ 2 at 3 months was considered a favorable functional outcome, while that of >2 was categorized as an unfavorable/poor functional outcome.

Other outcomes included new composite vascular events of recurrent IS, TIA, intracranial hemorrhage (ICH), and mortality during 12-month follow-up.

**Statistics**

Continuous variables are depicted as mean ± standard deviation. The numerical variable’s normality was elucidated *via* the Kolmogorov-Smirnov test. For comparing normally distributed variables, a two-tailed independent group t-test was applied. Variables which not normally distributed were analyzed by the Mann-Whitney test. For categorical variables, percentages were used, and a chi-square test was carried out for comparison. In cases of minimal predicted variable frequencies, Fisher's exact test was carried out. Univariate analysis methods were used to investigate patients' clinical data and baseline characteristics with and without END. Risk factors for END and poor 3-month outcome (mRS >2) were explored through multivariate logistic stepwise regression analysis. The receiver operating characteristic (ROC) curve was applied to assess the ability of sNfL to predict END and a poor 3-month outcome. Survival function estimates for clinical outcomes based on the level of sNfL were elucidated *via* the Kaplan-Meier curve and log-rank test. Survival curves were acquired at 12 months. P-value < 0.05 was termed statistically important.

**Results**

This investigation enrolled 319 acute IS patients; 89 (27.90%) of these suffered from END. Diabetes mellitus was more common, and clinical deficits were more severe on admission (initial NIHSS score, P=0.005), and the level of sNfL was higher (p=0.029) in patients with END than those without END according to univariate analysis (Supplemental Table 1). Furthermore, MRI/CT imaging revealed that WMLs were more severe (ARWMC Rating 2 and 3) in END patients (p<0.05). (Supplemental Table 1). The significant increase in sNfL expression is correlated with the severity of stroke. [NIHSS 1: sNFL median, 63.98 (IQR, 50.78–78.81) pg/mL; NIHSS 2–4: sNFL median, 67.45 (IQR, 51.70–83.65) pg/mL; NIHSS≥ 5: sNFL median, 73.87 (IQR, 61.06–88.58) pg/mL; ANOVA P=0.0067, Figure 2A]. No correlation was observed between the age of IS patients and the expression of sNfL [age<50: sNFL median, 70.71 (IQR,58.84–78.74); age 50-59: sNFL median, 67.18 (IQR,50.78–84.90); age 60-69: sNFL median, 62.95 (IQR,50.55–78.60); age ≥70: sNFL median, 66.57 (IQR,51.12–83.89); ANOVA P=0.3143, (Supplemental Figure 1). However, a marked rise was indicated in sNfL levels with severe WMLs than its absence [ARWMC scale 3: sNFL median,72.82 (IQR, 62.39–90.29) pg/mL; ARWMC 0: sNFL median, 60.41 (IQR, 46.84–72.73) pg/mL; p=0.0004] (Figure 2B). Moreover, there is no significant correlation between NfL levels and patients' history of prior atrial fibrillation, as well as the onset of new atrial fibrillation following hospital admission(p＞0.05).

Following multivariate analysis, several variables were determined to be closely linked with the incidence of END after IS, including initial NIHSS score[odds ratio (OR), 1.186, with a 95% confidence interval (CI), 1.069-1.317 (p=0.001)], sNfL (OR, 1.014; 95% CI, 1.001–1.028; P=0.040), Diabetes mellitus (OR, 2.160; 95% CI, 1.104-4.227, P=0.025), ARWMC scale 2 (OR, 3.587; 95% CI, 1.617-7.958, p=0.002) and ARWMC scale 3(OR, 3.075; 95% CI, 1.153-8.202, p=0.025) (Table 1). These findings indicate that a higher NIHSS score, severe white matter hyperintensities, diabetes mellitus, and upregulated sNfL are significant predictors of END after IS. After their discharge, all the patients were follow-up. The functional outcome of each patient was assessed at 3 months using the mRS score; those with an mRS score > 2 were considered to have a poor functional prognosis. Upon conducting univariate analysis, several factors were identified as potentially associated with poor outcome, including older age, elevated sNfL levels, lower GCS and higher NIHSS scores at admission, and the presence of white matter hyperintensities. These findings are summarized in Supplemental Table 2. The multivariate logistic regression results indicated that severe white matter hyperintensities, elevated sNfL, and a higher NIHSS score at admission were strongly correlated with poor functional outcomes (Table 2).

The ROC was employed using the sNfL for predicting END development odds within IS cases. The optimal threshold point was 53.40, its sensitivity was 83.1%, and its specificity was 36.5%. The AUC was 0.586 (95% CI, 0.519 - 0.653, p = 0.017). Moreover, the combination of variables including sNfL, initial NIHSS score and ARWMC rating increased the AUC ≤0.706(95% CI, 0.644–0.767; P<0.001). (Figure 3A). Through ROC analysis, the predictive value of sNfL on poor outcome at 3 months were analysis, the optimal threshold point was 62.09, its sensitivity was 63.6%, and its specificity was 51.1%. The AUC was 0.573(95% CI, 0.504 - 0.642, p = 0.044). Likewise, receiver operating curves was drawn for variables that remain valid after multivariate analysis, and the predictive value of combination of variables including sNfL, NIHSS score and ARWMC rating on poor outcome at 3 months were shown in Figure 3B, the AUC was 0.749 (95% CI, 0.690-0.809, P<0.001)

Test-characteristics of sNfL≥62.09 pg/mL based on the Youden-Index for prediction of poor functional outcome (mRS＞2) at 3-month after the index event. According to the Kaplan-Meier analysis, there was a markedly higher incidence of composite vascular events in participants with high sNfL levels during the 12-month follow-up (p= 0.0196), including recurrent IS, ICH and mortality (Figure 4).

**Discussion**

The reported (2016) lifetime risk of stroke in adults is approximately 25% globally[26]. END is a frequent comorbidity associated with acute IS, and its occurrence can have significant implications for both short-term outcomes, such as morbidity and death, and long-term recovery from stroke[17]. Although. The definition of END remains variable[16][27]; many studies have reported its relatively high incidence, from 12% to 42%[26]. This study defined END as an increase of 2 or more points in NIHSS score within a week after admission. Here, it was observed that the incidence of END was 27.9%. Moreover, it was indicated that sNfL, HNISS score, diabetes, severe white matter lesions (ARWMC Rating Scale 2 and 3) were independently related to the occurrence of END. Additionally, via ROC curve analysis, upregulated sNfL indicated significant predictive value for IS patients who experienced END. And showed a trend of independent improvement in AUC in joint diagnosis, highlighting its potential as a reliable predictive factor.

The NfL is a primary constituent and backbone of neurofilaments[9], essentially involved in axonal growth, stability, and intracellular transport of substances[4]. It has high specificity for neuronal injury and death. Under normal conditions, NfL remains relatively stable, with low levels in peripheral blood. However, during neuronal damage, its expression increase, making it a valuable biomarker for neuronal injury. Initially, we hypothesized that sNfL is a biomarker for neuronal axonal injury, with its serum levels associated with age and clinical deficits following IS. However, the obtained data indicates a correlation between sNfL levels and the clinical deficits during hospitalization (assessed by NIHSS), but is independent of the age of IS patients. This discovery is inconsistent with previous research results. There might be several reasons for this inconsistency; for instance, the Traenka *et al*. [28]findings were limited to cervical artery dissection patients, and blood samples were measured within 30 days. Marcis’ study included TIA patients and used a modified electrochemiluminescence (ECL) immunoassay, and blood samples was drawn upon admission or after 24 hours[29]. Moreover, Uphaus *et al*. [30]and Sellner *et al*. [31]also indicated different results on the correlation between neurofilament levels and the NIHSS score. However, Sellner *et al.* used neurofilament heavy chains for research. It is noteworthy that sNfL levels can be influenced by the blood collection timings[32]. From the data of this investigation, Nfl may reflect central nervous system injury, as its levels correlate with the severity of stroke (NIHSS score). Therefore, even in cases of transient ischemic attack and negative neuroimaging, Nfl levels may still offer evidence of central nervous system injury. This underscores its significance as an important adjunct for assessing and treating various types of stroke patients. While Nfl levels are correlated with age[7], our results indicate that in ischemic stroke patients, advanced age and atrial fibrillation does not show a significant correlation with Nfl levels.

This investigation categorized ARWMC into four levels based on Wahlund's research[25]. A substantial difference was indicated in sNfL levels between patients classified as severe white matter lesions (ARWMC Rating Scale 3) and those classified as no lesions (ARWMC Rating Scale 0). The data were consistent with previous findings, as a correlation was depicted between sNfL and white matter hyperintensities[13][33], suggesting that sNfL might act as an index for chronic and ongoing neuroaxonal damage. Furthermore, the binary logistic regression analysis discovered that severe white matter lesions (ARWMC Rating Scale 2 and 3) were independently associated with END.

Remarkably, it was found that up-regulated sNfL, initial NIHSS score, and ARWMC Rating Scale 2 and 3 were also independently related to the 3-month poor mRS score, consistent with the literature on IS[30][34].The ROC curve data indicated that while the sensitivity may be lower compared to the initial HNISS score prediction, the sNfL value can still be a reliable indicator for predicting a poor outcome at 3 months. Considering that sNfL depicts ongoing chronic axonal damage and the chronic progression of small vessel disease[32], the participants were followed up for 12 months. The follow-up endpoint events included recurrent IS, ICH, mortality. Kaplan-Meier analysis indicated a markedly higher hazard for endpoint within a median 12-month follow-up in patients with >62.09 pg/mL sNfL values, measured within 24 hours after the stroke, further supporting the notion that up-regulated sNfL indicate a greater likelihood for IS patients to experience a worsening of their early and long-term outcome symptoms.

There are several limitations to this study. First, END evaluation requires the exclusion of other factors that can increase NIHSS scores, such as hemorrhagic transformation, brain edema, or systemic diseases. Since patients with NIHSS scores > 10 upon admission were excluded, there may be a selection bias in the study population. Second, the 12-month follow-up period and the inclusion of various endpoint events, such as recurrent IS, ICH, and mortality, have also limited this investigation. Although we conducted a second sNfL measurement for patients experiencing cardiovascular events, there were instances where some patients did not undergo the second serum sNfL test, and the results were not available. Therefore, the association of immediate sNfL levels with recurrent cardiovascular events remains unclear.

**Conclusions**

Overall, the acute IS patients indicated a high occurrence of END. Furthermore, higher initial NIHSS score, severe white matter hyperintensities, upregulated sNfL, and the presence of diabetes was independently linked with END. Additionally, a higher initial NIHSS score, severe white matter hyperintensities, and upregulated sNfL were also independent variables linked with poor functional outcomes at 3 months. The data indicated that sNfL could be a reliable indicator for predicting END and poor functional outcomes and might also help identify individuals with an enhanced risk for cardiovascular events, making it a potential predictor for future therapeutic trials.

**Acknowledgements**

**Funding**

This study was supported by the General Program of the National Natural Science Foundation of China (grant numbers 82173175), Knowledge Innovation Program of the Chinese Academy of Sciences (grant numbers JH2022007) and 1·3·5 project for disciplines of excellence–Clinical Research Incubation Project, West China Hospital, Sichuan University (grant numbers 2020HXFH036). National Natural Science Foundation of China (grant number 82001320). Key R&D projects in Sichuan Province (2022YFS0319)

**Competing Interests**

The authors have no relevant financial or non-financial interests to disclose

**Author Contributions**

Conceptualization, XCX, QT, XJG and YT.; methodology, XCX, YT; software, XCX QT; validation, QXY, JYL and MJP.; formal analysis, XCX and QT.; investigation, XCX; resources, QT, YT and YXY.; data curation, YXY.; writing—original draft preparation, XCX.; writing—review and editing, MJP; visualization, XCX; supervision, MJP; project administration, XJG and MJP; funding acquisition, MJP and XJG. All authors have read and agreed to the published version of the manuscript.

**Data Availability**

All data generated or analysed during this study are included in this published article [and its supplementary information files]

**Ethics approval**

This study complied with the guidelines for human studies and was conducted ethically in accord-ance with the World Medical Association Declaration of Helsinki. The study protocol was reviewed and approved by the Ethic Committee of the Deyang People’s Hospital, approval number (2019-04-142-k01).

**Consent to participate**

Informed consent was obtained from all individual participants included in the study.

**Reference**

1. Feigin VL, Lawes CM, Bennett DA, Barker-Collo SL, Parag V. Worldwide stroke incidence and early case fatality reported in 56 population-based studies: a systematic review. Lancet Neurol. (2009) 8:355–69. doi: 10.1016/S1474-4422(09)70025-0

2. Wang YJ, Li ZX, Gu HQ, Zhai Y, Zhou Q, Jiang Y, et al. China stroke statistics: an update on the 2019 report from the National Center for healthcare quality Management in Neurological Diseases, China National Clinical Research Center for neurological diseases, the Chinese Stroke Association, National Center f. Stroke Vasc Neurol. (2022) 7:415–50. doi: 10.1136/svn-2021-001374.

3. Foerch C, Singer OC, Neumann-Haefelin T, du Mesnil de Rochemont R, Steinmetz H, Sitzer M. Evaluation of serum S100B as a surrogate marker for long-term outcome and infarct volume in acute middle cerebral artery infarction. Arch Neurol. (2005) 62:1130–4. doi: 10.1001/archneur.62.7.1130.

4. Gaetani L, Blennow K, Calabresi P, Di Filippo M, Parnetti L, Zetterberg H. Neurofilament light chain as a biomarker in neurological disorders. J Neurol Neurosurg Psychiatry. (2019) 90:870–81. doi: 10.1136/jnnp-2018-320106

5. Bridel C, van Wieringen WN, Zetterberg H, Tijms BM, Teunissen CE, AlvarezCermeño JC, et al. Diagnostic value of cerebrospinal fluid Neurofilament light protein in neurology: a systematic review and Meta-analysis. JAMA Neurol. (2019) 76:1035–48. doi: 10.1001/jamaneurol.2019.1534.

6. Khalil M, Pirpamer L, Hofer E, Voortman MM, Barro C, Leppert D, et al. Serum neurofilament light levels in normal aging and their association with morphologic brain changes. Nat Commun. (2020) 11:812. doi: 10.1038/s41467-020-14612-6

7. Abu-Rumeileh S, Abdelhak A, Foschi M, D'Anna L, Russo M, Steinacker P, et al. The multifaceted role of neurofilament light chain protein in non-primary neurological diseases. Brain. (2023) 146:421–37. doi: 10.1093/brain/awac328

8. Abdelhak A, Barba L, Romoli M, Benkert P, Conversi F, D’Anna L, et al. Prognostic performance of blood neurofilament light chain protein in hospitalized COVID-19 patients without major central nervous system manifestations: an individual participant data meta-analysis. J Neurol. (2023) 270:3315–28. doi: 10.1007/s00415-023-11768-1

9. Lee Y, Lee BH, Yip W, Chou P, Yip B-S. Neurofilament proteins as prognostic biomarkers in neurological disorders. Curr Pharm Des. (2020) 25:4560–9. doi: 10.2174/1381612825666191210154535

10. Mattsson N, Andreasson U, Zetterberg H, Blennow K, Aisen P, et al. Association of plasma neurofilament light with neurodegeneration in patients with Alzheimer disease. JAMA Neurol. (2017) 74:557–66. doi: 10.1001/jamaneurol.2016.6117.

11. Disanto G, Barro C, Benkert P, Naegelin Y, Schädelin S, Giardiello A, et al. Serum Neurofilament light: a biomarker of neuronal damage in multiple sclerosis. Ann Neurol. (2017) 81:857–70. doi: 10.1002/ana.24954.

12. Poesen K, de Schaepdryver M, Stubendorff B, Gille B, Muckova P, Wendler S, et al. Neurofilament markers for ALS correlate with extent of upper and lower motor neuron disease. Neurology. (2017) 88:2302–9. doi: 10.1212/WNL.0000000000004029

13. Tiedt S, Duering M, Barro C, Kaya AG, Boeck J, Bode FJ, et al. Serum neurofilament light a biomarker of neuroaxonal injury after ischemic stroke. Neurology. (2018) 91:E1338–47. doi: 10.1212/WNL.0000000000006282.

14. Weimar C, Mieck T, Buchthal J, Ehrenfeld CE, Schmid E, Diener HC, et al. Neurologic worsening during the acute phase of ischemic stroke. Arch Neurol. (2005) 62:393–7. doi: 10.1001/archneur.62.3.393.

15. Birschel P, Ellul J, Barer D. Progressing stroke: towards an internationally agreed

definition. Cerebrovasc Dis. (2004) 17:242–52. doi: 10.1159/000076161.

16. Tei H, Uchiyama S, Ohara K, Kobayashi M. Deteriorating ischemic stroke in 4 clinical categories classified by the Oxfordshire community stroke project. Stroke. (2000) 31:2049–54. doi: 10.1161/01.str.31.9.2049.

17. Thanvi B, Treadwell S, Robinson T. Early neurological deterioration in acute ischaemic stroke: Predictors, mechanisms and management. Postgrad Med J. (2008) 84:412–8. doi: 10.1136/pgmj.2007.066118

18. Seners P, Baron JC. Revisiting ‘progressive stroke’: incidence, predictors, pathophysiology, and management of unexplained early neurological deterioration following acute ischemic stroke. J Neurol. (2018) 265:216–25. doi: 10.1007/ s00415-017-8490-3.

19. Yilmaz A, Blennow K, Hagberg L, Nilsson S, Price RW, Schouten J, et al. Neurofilament light chain protein as a marker of neuronal injury: review of its use in HIV-1 infection and reference values for HIV-negative controls. Expert Rev Mol Diagn. (2017) 17:761–70. doi: 10.1080/14737159.2017.1341313

20. Xu C, Xu H, Yi T, Yi X, Ma J. Cerebral microbleed burden in ischemic stroke patients on aspirin: prospective cohort of intracranial hemorrhage. Front Neurol. (2021) 12:1–7. doi: 10.3389/fneur.2021.742899

21. Liu Y, Zhang M, Bao H, Zhang Z, Mei Y, Yun W, et al. The efficacy of intravenous thrombolysis in acute ischemic stroke patients with white matter hyperintensity. Brain Behav. (2018) 8:1–9. doi: 10.1002/brb3.1149.

22. Lau KK, Li L, Schulz U, Simoni M, Chan KH, Ho SL, et al. Total small vessel disease score and risk of recurrent stroke. Neurology. (2017) 88:2260–7. doi: 10.1212/ WNL.0000000000004042.

23. Li J, Zhang P, Zhu Y, Duan Y, Liu S, Fan J, et al. Serum neurofilament light chain levels are associated with early neurological deterioration in minor ischemic stroke. Front Neurol. (2023) 14:14. doi: 10.3389/fneur.2023.1096358.

24. O'Connell GC, Alder ML, Smothers CG, Still CH, Webel AR, Moore SM. Diagnosis of ischemic stroke using circulating levels of brain-specific proteins measured via high-sensitivity digital ELISA. Brain Res. (2020) 1739:146861. doi: 10.1016/j. brainres.2020.146861

25. Wahlund LO, Barkhof F, Fazekas F, Bronge L, Augustin M, Sjögren M, et al. A new rating scale for age-related white matter changes applicable to MRI and CT. Stroke. (2001) 32:1318–22. doi: 10.1161/01.STR.32.6.1318.

26. GBD 2016 Lifetime Risk of Stroke CollaboratorsFeigin VL, Nguyen G, Cercy K, Johnson CO, Alam T, et al. Global, regional, and country-specific lifetime risks of stroke, 1990 and 2016. N Engl J Med. (2018) 379:2429–37. doi: 10.1056/NEJMoa1804492

27. Da A, Toni D, Iweins F, Lesaffre E, Bastianello S. Potential predictors and associated factors in the European cooperative. Stroke. (1999):2631–6.

28. Traenka C, Disanto G, Seiffge DJ, Gensicke H, Hert L, Grond-Ginsbach C, et al. Serum Neurofilament light chain levels are associated with clinical characteristics and outcome in patients with cervical artery dissection. Cerebrovasc Dis. (2015) 40:222–7. doi: 10.1159/000440774

29. De Marchis GM, Katan M, Barro C, Fladt J, Traenka C, Seiffge DJ, et al. Serum neurofilament light chain in patients with acute cerebrovascular events. Eur J Neurol. (2018) 25:562–8. doi: 10.1111/ene.13554.

30. Uphaus T, Bittner S, Gröschel S, Steffen F, Muthuraman M, Wasser K, et al. NfL (Neurofilament light chain) levels as a predictive marker for long-term outcome after ischemic stroke. Stroke. (2019) 50:3077–84. doi: 10.1161/STROKEAHA.119.026410.

31. Sellner J, Patel A, Dassan P, Brown MM, Petzold A. Hyperacute detection of neurofilament heavy chain in serum following stroke: a transient sign. Neurochem Res. (2011) 36:2287–91. doi: 10.1007/s11064-011-0553-8.

32. chreiber S, Scheumann V, Perosa V, Vielhaber S, Assmann A. Reader response: serum neurofilament light is sensitive to active cerebral small vessel disease. Neurology. (2018) 90:1126. doi: 10.1212/WNL.0000000000005672.

33. Jonsson M, Zetterberg H, Van Straaten E, Lind K, Syversen S, Edman Å, et al. Cerebrospinal fluid biomarkers of white matter lesions—cross-sectional results from the LADIS study. Eur J Neurol. (2010) 17:377–82. doi: 10.1111/j.1468-1331.2009.02808.x

34. Debette S, Markus HS. The clinical importance of white matter hyperintensities on brain magnetic resonance imaging: systematic review and meta-analysis. BMJ. (2010) 341:c3666. doi: 10.1136/bmj.c3666

**Figure Captions**

**Figure 1:** A flowchart depicting participant’s enrollment/follow-up.

**Figure 2: A:** Stratified analysis elucidated the association of sNfL (serum neurofilament light chain) levels with stroke severity (NIHSS score). **B:** Stratified analysis evaluated the relationship between sNfL levels and age-related white matter changes. *P value <0.05.

**Figure 3: A:** ROC to predict the early neurological deterioration for the combination of diabetes mellitus, National Institutes of Health Stroke Scale (NIHSS), and ARMWC-rating (blue) exhibited an area under the curve (AUC) of 0.694 (95% CI, 0.631–0.756; P<0.001). The additional use of sNfL (red) showed a trend toward an independent improvement of AUC to 0.706 (95% CI, 0.644–0.767; P<0.001). **B:** ROC to predict the 3 months poor outcome for the combination of National Institutes of Health Stroke Scale (NIHSS) and ARMWC-rating (blue) exhibited an area under the curve (AUC) of 0.740 (95% CI, 0.678–0.801; P<0.001). The additional use of sNfL (red) showed a trend toward an independent improvement of AUC to 0.749 (95% CI, 0.690–0.809; P<0.001).

**Figure 4:** The time to cardiovascular endpoint (recurrent IS, intracranial hemorrhage, and mortality) was analyzed according to the division of patients using the Youden index based on the sNfL level’s ROC curve analysis. Patients with high sNfL levels (≥62.09 pg/mL) are represented in red, while those with low sNfL levels (<62.09 pg/mL) are represented in blue. The analysis was performed using non-logarithmic-transformed values, and the intergroup differences were assessed *via* the log-rank test. The data indicated markedly significant intergroup differences (P=0.0196), indicating that patients with high sNfL levels had a shorter time to reach the cardiovascular endpoint than those with low NfL levels.

**Supplementary Materials:**

**Supplemental methods**

**Supplemental Table 1**. Characteristics of individuals with and without END after IS.

**Supplemental Table 2.** Characteristics of individuals about their functional outcome 90 d after IS.

**Supplemental Figure 1:** Stratified analysis was conducted to evaluate the relationship between sNfL (neurofilament light chain) levels and age.

| Variable | OR | 95%CI | P value |
| --- | --- | --- | --- |
| Diabetes mellitus | 2.160 | 1.104-4.227 | 0.025* |
| Serum NfL value | 1.014 | 1.001-1.028 | 0.040* |
| Blood glucose at admission | 1.043 | 0.980-1.110 | 0.187 |
| NIHSS score at admission | 1.186 | 1.069-1.317 | 0.001* |
| ARWMC（2） | 3.587 | 1.617-7.958 | 0.002* |
| ARWMC（3） | 3.075 | 1.153-8.202 | 0.025* |

**Table 1. Multivariate logistic regression analysis with regard to the early neurological deterioration after stroke.**

**ARWMC (Age-Related White Matter Changes) Rating Scale 2: Beginning confluence of lesions); ARWMC Rating Scale 3: diffuse involvement of the entire region, with or without involvement of U fibers.** ***P value <0.05.**

| Variable | OR | 95%CI | P value |
| --- | --- | --- | --- |
| Age | 1.026 | 0.999-1.053 | 0.059 |
| Serum NfL value | 1.015 | 1.001-1.029 | 0.038* |
| GCS score at admission | 0.938 | 0.780-1.129 | 0.501 |
| NIHSS score at admission | 1.271 | 1.136-1.422 | ＜0.001* |
| ARWMC（2） | 3.038 | 1.333-6.924 | 0.008* |
| ARWMC（3） | 4.424 | 1.661-11.781 | 0.003* |

**Table 2. Multiple Logistic regression with three-month poor functional outcome（mRS＞2）**

**ARWMC (Age-Related White Matter Changes) Rating Scale 2: Beginning confluence of lesions); ARWMC Rating Scale 3: diffuse involvement of the entire region, with or without involvement of U fibers. *P value <0.05.**
