# Supplementary material for: Serum neurofilament light chain: a predictive marker for outcomes following mild-to-moderate ischemic stroke
Source: Front Neurol. 2024 May 22;15:1398826. doi: 10.3389/fneur.2024.1398826 (PMC11150679; doi:10.3389/fneur.2024.1398826)
Supplement: Supplementary file 3 [file Table_1.docx]

**Supplemental Table 1. Characteristics of individuals with and without early neurological deterioration after ischemic stroke.**

| variable | No END | END | p-value |
| --- | --- | --- | --- |
| N（%） | 230（72.10） | 89（27.90） |  |
| Age，y | 67.90（±11.43） | 67.53（±11.90） | 0.795 |
| Sex（male, %） | 150（65.22） | 53（59.55） | 0.345 |
| Time from onset to admission（h） | 17.29（±26.32） | 19.71（±30.16） | 0.480 |
| History of ischemic stroke （n，%） | 38（16.52） | 14（15.73） | 0.864 |
| Using antiplatelet drugs before onset | 28（12.17） | 9（10.11） | 0.606 |
| Smoking (current/former) | 123（53.48） | 39（43.82） | 0.122 |
| Drinking (current/former) | 112（48.70） | 41（46.07） | 0.673 |
| Hypertension | 147（63.91） | 58（65.16） | 0.834 |
| Diabetes mellitus | 47（20.43） | 31（34.83） | 0.007* |
| Dyslipidemia | 66（30.28） | 28（32.18） | 0.744 |
| Previous atrial fibrillation | 15（6.52） | 7（7.87） | 0.671 |
| New onset atrial fibrillation | 20（8.73） | 5（5.62） | 0.354 |
| Coronary heart disease | 20（8.70） | 6（6.74） | 0.567 |
| Serum NfL value（pg/mL） | 64.46（±20.00） | 69.87（±19.00） | 0.029* |
| Systolic blood pressure at admission（mmHg） | 155.02（±23.94） | 160.35（±24.13） | 0.076 |
| Diastolic blood pressure at admission（mmHg） | 87.80（±13.87） | 90.10（±13.78） | 0.185 |
| Blood glucose at admission（mmol/L） | 8.60（±4.16） | 9.98（±5.05） | 0.023* |
| GCS score at admission | 14.66（±1.25） | 14.31（±1.51） | 0.060 |
| NIHSS score at admission | 3.57（±2.40） | 4.44（±2.59） | 0.005* |
| **Stroke territory** |  |  |  |
| Anterior cerebral circulation (%) | 177（76.96） | 64（71.91） | 0.347 |
| Posterior cerebral circulation (%) | 53（23.04） | 25（28.09） | 0.347 |
| **Treatment:** |  |  |  |
| Using aspirin alone or clopidogrel alone | 83 (36.09) | 37 (41.57) | 0.364 |
| Dual antiplatelet therapy | 122 (53.04) | 38 (42.70) | 0.097 |
| Anticoagulation | 25 (10.97) | 14 (15.73) | 0.235 |
| Thrombolysis | 42（18.26） | 21 (23.60) | 0.283 |
| **ARWMC Rating Scale** |  |  | 0.038* |
| 0 | 73（31.74） | 18（20.22） |  |
| 1 | 82（35.65） | 27（30.34） |  |
| 2 | 52（22.61） | 31（34.83） |  |
| 3 | 23（10.00） | 13（14.61） |  |
| **New subtype classification** |  |  | 0.632 |
| ASLA (%) | 112 (48.70) | 46 (51.69) |  |
| SAD (%) | 40 (17.39) | 23(25.84) |  |
| CE (%) | 51(22.17) | 8 (8.99) |  |
| SUD (%) | 27 (11.74) | 12 (13.48) |  |

**Abbreviations: ASLA, atherothrombosis with significant stenosis of a large artery; CE, cardioembolism; SAD, small artery disease; SP, stroke progression; SUD,**

**stroke of undetermined etiology. *p < .05 was considered statistically significant.**
