# Supplementary material for: Serum neurofilament light chain: a predictive marker for outcomes following mild-to-moderate ischemic stroke
Source: Front Neurol. 2024 May 22;15:1398826. doi: 10.3389/fneur.2024.1398826 (PMC11150679; doi:10.3389/fneur.2024.1398826)
Supplement: Supplementary file 4 [file Table_2.docx]

**Supplemental Table 2. Characteristics of individuals with regard to their functional**

**outcome 90 d after ischemic stroke.**

| variable | mRS≤2 | mRS＞2 | p-value |
| --- | --- | --- | --- |
| n（%） | 231（72.41） | 88（27.59） |  |
| Age（y） | 66.41（±11.24） | 71.83（11.24） | ＜0.001* |
| Sex（male, %） | 149（64.50） | 53（60.23） | 0.554 |
| Time from onset to admission（h） | 17.40（±24.00） | 19.39（±35.14） | 0.565 |
| Serum NfL value（pg/mL） | 64.62（±19.82） | 69.60（±19.66） | 0.046* |
| Systolic blood pressure at admission（mmHg） | 155.96（±23.51） | 157.99（±25.72） | 0.505 |
| Diastolic blood pressure at admission（mmHg） | 89.38（±13.75） | 85.99（±14.01） | 0.052 |
| Blood glucose at admission（mmol/L） | 8.99（±4.55） | 8.99（±4.27） | 0.998 |
| GCS score at admission | 14.67（±1.25） | 14.28（±1.51） | 0.036* |
| NIHSS score at admission | 3.43（±2.30） | 4.80（±2.67） | ＜0.001* |
| History of stroke（n，%） | 37（16.02） | 19（21.59） | 0.224 |
| History of cerebral infarction（n，%） | 33（14.29） | 19（21.59） | 0.104 |
| Using antiplatelet drugs before onset | 27（11.69） | 10（11.36） | 0.962 |
| Smoking (current/former) | 116（50.22） | 45（51.14） | 0.811 |
| Drinking (current/former) | 114（49.35） | 38（43.18） | 0.367 |
| Hypertension | 146（63.20） | 59（67.05） | 0.444 |
| Diabetes mellitus | 54（23.38） | 24（27.27） | 0.437 |
| Dyslipidemia | 71（30.73） | 23（26.14） | 0.458 |
| Previous atrial fibrillation | 13（5.62） | 8（9.09） | 0.253 |
| New onset atrial fibrillation | 15（6.49） | 9（10.22） | 0.251 |
| Coronary heart disease | 17（7.36） | 9（10.22） | 0.386 |
| Site of stroke occurrence |  |  |  |
| Internal carotid artery system | 174（75.32） | 67（76.14） | 0.754 |
| Vertebrobasilar artery system | 60（25.97） | 20（22.73） | 0.584 |
| Cardiogenic stroke | 29（12.55） | 16（18.18） | 0.183 |
| Thrombolysis | 42（18.18） | 21（23.86） | 0.235 |
| ARWMC Rating Scale |  |  | ＜0.001* |
| 0 | 79（34.35） | 12（13.64） |  |
| 1 | 83（35.93） | 27（30.68） |  |
| 2 | 51（22.08） | 31（35.23） |  |
| 3 | 18（7.79） | 18（20.45） |  |

***P value <0.05.**
